# Resting-state functional connectivity in children cooled for neonatal encephalopathy

**DOI:** 10.1093/braincomms/fcae154

**Published:** 2024-04-29

**Authors:** Arthur P C Spencer, Marc Goodfellow, Ela Chakkarapani, Jonathan C W Brooks

**Affiliations:** Clinical Research and Imaging Centre, University of Bristol, Bristol BS2 8DX, UK; Translational Health Sciences, Bristol Medical School, University of Bristol, Bristol BS8 1TH, UK; Department of Radiology, Lausanne University Hospital, 1011 Lausanne, Switzerland; Living Systems Institute, University of Exeter, Exeter EX4 4QD, UK; Department of Mathematics and Statistics, University of Exeter, Exeter EX4 4QF, UK; Translational Health Sciences, Bristol Medical School, University of Bristol, Bristol BS8 1TH, UK; Neonatal Intensive Care Unit, St Michaels Hospital, University Hospitals Bristol and Weston NHS Foundation Trust, Bristol BS2 8EG, UK; Clinical Research and Imaging Centre, University of Bristol, Bristol BS2 8DX, UK; University of East Anglia Wellcome Wolfson Brain Imaging Centre (UWWBIC), University of East Anglia, Norwich NR4 7TJ, UK

**Keywords:** hypoxic-ischaemic encephalopathy, resting-state functional connectivity, dynamic functional connectivity, fMRI

## Abstract

Therapeutic hypothermia improves outcomes following neonatal hypoxic-ischaemic encephalopathy, reducing cases of death and severe disability such as cerebral palsy compared with normothermia management. However, when cooled children reach early school-age, they have cognitive and motor impairments which are associated with underlying alterations to brain structure and white matter connectivity. It is unknown whether these differences in structural connectivity are associated with differences in functional connectivity between cooled children and healthy controls. Resting-state functional MRI has been used to characterize static and dynamic functional connectivity in children, both with typical development and those with neurodevelopmental disorders. Previous studies of resting-state brain networks in children with hypoxic-ischaemic encephalopathy have focussed on the neonatal period. In this study, we used resting-state fMRI to investigate static and dynamic functional connectivity in children aged 6–8 years who were cooled for neonatal hypoxic-ischaemic without cerebral palsy [*n* = 22, median age (interquartile range) 7.08 (6.85–7.52) years] and healthy controls matched for age, sex and socioeconomic status [*n* = 20, median age (interquartile range) 6.75 (6.48–7.25) years]. Using group independent component analysis, we identified 31 intrinsic functional connectivity networks consistent with those previously reported in children and adults. We found no case-control differences in the spatial maps of these intrinsic connectivity networks. We constructed subject-specific static functional connectivity networks by measuring pairwise Pearson correlations between component time courses and found no case-control differences in functional connectivity after false discovery rate correction. To study the time-varying organization of resting-state networks, we used sliding window correlations and deep clustering to investigate dynamic functional connectivity characteristics. We found *k* = 4 repetitively occurring functional connectivity states, which exhibited no case-control differences in dwell time, fractional occupancy or state functional connectivity matrices. In this small cohort, the spatiotemporal characteristics of resting-state brain networks in cooled children without severe disability were too subtle to be differentiated from healthy controls at early school-age, despite underlying differences in brain structure and white matter connectivity, possibly reflecting a level of recovery of healthy resting-state brain function. To our knowledge, this is the first study to investigate resting-state functional connectivity in children with hypoxic-ischaemic encephalopathy beyond the neonatal period and the first to investigate dynamic functional connectivity in any children with hypoxic-ischaemic encephalopathy.

## Teaser

Spencer et al. assessed static and dynamic resting-state functional connectivity in children treated with therapeutic hypothermia for neonatal encephalopathy, compared with healthy controls. They report no case-control differences in resting-state network spatial maps or in static or dynamic functional connectivity properties, despite previously reporting structural connectivity differences in this cohort.

## Graphical Abstract

**Graphical abstract:**
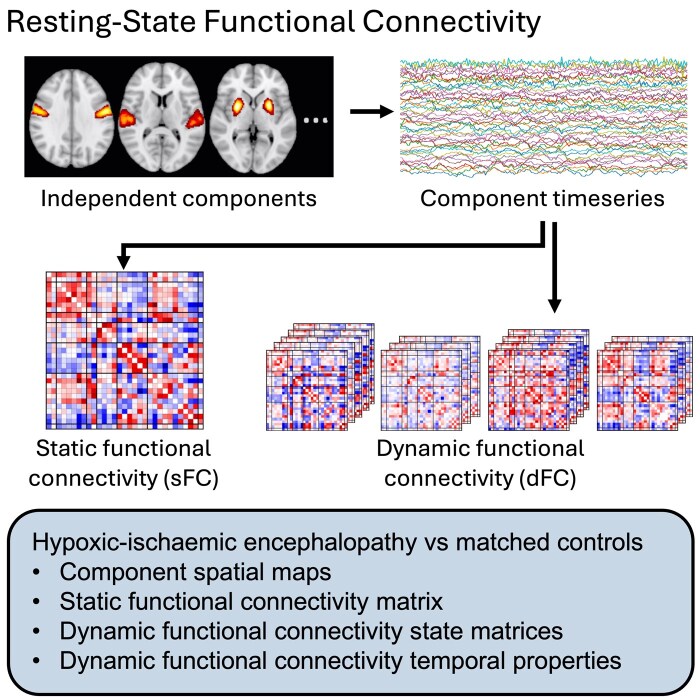

## Introduction

Therapeutic hypothermia has considerably improved outcomes following neonatal hypoxic-ischaemic encephalopathy (HIE) secondary to perinatal asphyxia. Cooled infants are at reduced risk of death or severe disability, such as cerebral palsy, compared with normothermia management following HIE.^1-3^ Therapeutic hypothermia is therefore standard care for HIE in most high-income counties. However, despite the benefits of therapeutic hypothermia, there are still aspects of brain development which are impacted by HIE. At early school-age, children cooled for HIE, who do not have cerebral palsy, have cognitive and motor impairments,^4,5^ attention and visuospatial processing difficulties^6^ and communication difficulties^7^ compared with healthy controls. An understanding of the differences in brain structure and function between cooled children and healthy controls is required to inform research into therapeutic intervention strategies to promote healthy brain development.

Functional MRI (fMRI) allows non-invasive investigation of brain activity by measuring changes in the blood oxygen level-dependent (BOLD) signal. In resting-state fMRI, the participant is scanned during rest (i.e. without engaging in a task or responding to stimuli) in order to measure spontaneous fluctuations in the BOLD signal.^8^ Functional connectivity (FC) analysis allows investigation of functional interactions across the brain, by measuring correlations between pairs of brain regions in these low-frequency fluctuations of recorded BOLD signal.^9-11^ This approach can be extended to study the time-varying organization of resting-state brain activity using dynamic functional connectivity (dFC) analysis.^12-15^ One such approach is to use sliding window correlations to calculate a series of FC matrices for each subject, which can then be clustered at the group level, revealing brain states representing repetitively occurring FC patterns.^12,16^ Spatiotemporal characteristics of these dFC states have been characterized in typically developing children^17,18^ and have been shown to be sensitive to neurodevelopmental outcomes.^17,19-23^

Studies of neonates with HIE (including both those with and without severe disability such as cerebral palsy) have found alterations to resting-state FC compared with healthy controls^24,25^ and associations between FC and both HIE severity and motor and developmental assessment scores.^26-28^ However, it remains unclear whether brain function in this population is affected in later life. We have previously shown that children cooled for HIE have disrupted white matter connectivity^29-31^ and structural alterations to subcortical structures^32^ and mammillary bodies^33^ compared with healthy controls at early school-age. It is unknown whether these alterations to brain structure and structural connectivity are associated with measurable differences in functional brain activity in these children.

Given that previous studies have shown that HIE severity is associated with outcomes and altered resting-state activity in neonates, we assessed whether resting-state FC following moderate to severe neonatal HIE differed from healthy controls at 6–8 years of age, in the absence of severe motor disability. We investigated resting-state brain activity using fMRI in children aged 6–8 years without cerebral palsy who were treated with therapeutic hypothermia for neonatal HIE (cases) and healthy controls matched for age, sex and socioeconomic status. We used group-level independent component analysis (ICA) to determine a set of intrinsic connectivity networks (ICNs) and then studied case-control differences in the spatial maps of these ICNs and in static and dynamic FC between ICNs. To our knowledge, this is the first study to investigate FC in children with HIE beyond the neonatal period and the first study to investigate dFC in any children with HIE.

## Materials and methods

### Participants

This study investigated participants of the ‘CoolMRI’ study,^5,29^ a study of early school-age children without cerebral palsy who received therapeutic hypothermia as a neuroprotective intervention for neonatal HIE and control children matched for age, sex and socioeconomic status. Informed and written consent was obtained from the parents of participants and assent obtained from the children. Ethical approval was obtained from the North Bristol Research Ethics Committee and the Health Research Authority (REC ID: 15/SW/0148).

Cases were aged 6–8 years and were sequentially selected from those who received therapeutic hypothermia between October 2007 and November 2012 for moderate to severe encephalopathy, confirmed by amplitude-integrated EEG assessment,^2^ secondary to perinatal asphyxia. Cases did not have a diagnosis of cerebral palsy at 2 and at 6–8 years based on neurological examination and assessment of motor function. Children were excluded if they were cooled outside the standard criteria, born before 35 weeks gestation, had any additional diagnosis apart from HIE (such as genetic or metabolic disorder), had a major intracranial haemorrhage or congenital brain malformation visible on neonatal MRI or were non-native English speakers.

Age-, sex- and socioeconomic status-matched controls were recruited through local schools and newsletters circulated at the University of Bristol. Children were included who were born at >35 weeks gestation, had not had perinatal asphyxia with HIE and spoke English as their primary spoken language.

Socioeconomic status was measured based on participant’s postcode at examination, using the index of multiple deprivation as defined for England by the UK Government (www.gov.uk/government/statistics/english-indices-of-deprivation-2019). Each postcode in England is assigned a number, on a scale of 1–10, indicating the decile within which the local area is ranked in the country, from most deprived (1) to least deprived (10).

### MRI acquisition

Images were acquired using a 3 Tesla Siemens Magnetom Skyra and a 32-channel receive-only head coil. A child-friendly, detailed explanatory video was sent to the family before assessment day and presented again on the day of the scan together with the typical sounds in the MRI scanner. Head movement was minimized using cushions. A T_1_-weighted volumetric scan was obtained, for spatial normalization, with a magnetization-prepared rapid acquisition gradient echo (MPRAGE) pulse sequence using the following parameters: echo time (TE) = 2.19 ms, inversion time (TI) = 800 ms, repetition time (TR) = 1500 ms, flip angle = 9°, field of view = 234 × 250 mm, 176 slices, 1.0 mm isotropic voxels and generalized autocalibrating partially parallel acquisitions (GRAPPA) acceleration factor 4.^34^ During acquisition of the volumetric scan, a film of the participants’ choice was projected onto a screen visible through the mirror assembly of the head coil. During the resting-state functional acquisition, the film was turned off and participants were instructed to keep their eyes open and look at a central fixation cross. T_2_-weighted functional images were acquired using a gradient echo planar imaging sequence with the following parameters: TE = 30 ms, TR = 906 ms, multiband factor 6, flip angle = 60°, field of view = 185 × 185 mm, matrix = 64 × 64, slice thickness = 3.125 mm, 36 slices and 2.890 × 2.890 × 3.125 mm voxels. We acquired 300 volumes giving a scan time of 4 min 32 s. We also acquired dual-(gradient)-echo images for distortion correction of fMRI data (see below).

### Preprocessing

Resting-state fMRI data were preprocessed using FEAT^35^ from the FMRIB Software Library (FSL v6.0, https://fsl.fmrib.ox.ac.uk).^36,37^ Processing steps were as follows: (i) the first 5 volumes in the sequence were discarded to ensure steady-state magnetization, leaving 295 volumes (4 minutes 27 seconds); (ii) motion correction was then applied with MCFLIRT^38^ to align all volumes in the sequence using rigid-body registration; (iii) the derived fieldmap was used to correct distortions (induced by magnetic field inhomogeneities) in the fMRI data; (iv) non-brain tissue was removed using brain extraction tool (BET); (v) spatial smoothing was performed with a 5 mm full-width at half-maximum Gaussian kernel; and (vi) high-pass temporal filtering was applied with a cut-off of 150 s to remove low-frequency artefacts. Preprocessed fMRI data were then transformed to Montreal Neurological Institute (MNI) standard space; first, subject fMRI data were registered to the subject’s T_1_-weighted image using rigid-body registration, and then, the subject T_1_-weighted image was registered to the MNI standard template using nonlinear registration, and the resulting transformation was applied to the fMRI data.

Following standard preprocessing steps, each subject’s fMRI data were cleaned to remove artefacts due to motion, physiological noise and scanner noise, using FSL’s FIX.^39,40^ FIX uses a training data set to automatically classify subject-level ICA components (calculated using MELODIC from FSL) into signal and noise and then regresses out the noise components from the fMRI data. A study-specific training data set was generated by hand-labelling components from a random sample of 15 subjects which were matched to the full cohort for case-control status. For each subject in the training sample, components were labelled signal or noise by two raters (A.P.C.S. and J.C.W.B.) based on characteristics of the spatial maps, timeseries and frequency spectra (for detailed description of characteristics of signal and noise components, see Griffanti *et al.*^41^). Leave-one-out cross-validation of the training data set gave a mean true positive rate of 94.2% and a mean true negative rate of 88.4%. The training data set was used to denoise all subjects’ fMRI data, including regressing out the movement parameters estimated during the motion correction preprocessing step.

### Quality control

To assess quality of the fMRI scan, we quantified the amount of movement of each subject during acquisition using mean framewise displacement and maximum absolute displacement. Framewise displacement combines measurements of translation (*x*, *y*, *z*) and rotation (pitch, yaw, roll) into a single scalar quantity to summarize instantaneous head motion at each timepoint. This was calculated according to Power *et al.*,^42^ using the movement parameters estimated during the motion correction preprocessing step, and averaged across timepoints to give mean framewise displacement for each subject. Note that this is likely an overestimation of the framewise displacement, as rotational displacements are calculated based on an approximate radius (distance from the centre of the brain to the cortex) of 50 mm, but this distance will be slightly smaller in this paediatric cohort. Acquisitions were excluded if they had a mean framewise displacement > 0.5 or if the maximum absolute displacement from the reference volume exceeded 4 mm. T_1_-weighted scans were visually assessed, and those with severe movement artefact, which would affect the registration of the subject data to the standard template, were excluded.

### Group independent component analysis

Following preprocessing, resting-state fMRI data for the whole cohort were analysed using spatial group ICA (GICA). GICA decomposes data into maximally spatially independent components, whose time courses can be linearly combined to reconstruct the original data. GICA was applied using GIFT,^43,44^ as follows. An initial dimensionality reduction step was applied to the fMRI data for each subject, using principal component analysis (PCA) to reduce 295 timepoint data to 120 directions of maximal variability. Subject data for the whole cohort were then concatenated across time, and a group PCA step reduced this into 100 components with the expectation maximization algorithm. The infomax algorithm^45^ was then used to calculate 100 independent components from the reduced-dimensionality group data. To ensure robust estimation of independent components, ICA was repeated 20 times using ICASSO, and aggregate spatial maps were estimated as the modes of component clusters. We selected only components which gave a stability index (*I*_q_) > 0.8 in ICASSO. For these components, subject-specific spatial maps and time courses were calculated using the GICA back-reconstruction method, which is analogous to dual regression, differing only in the projection through the initial PCA step.^44^

We inspected the spatial maps and temporal properties of the independent components to identify ICNs based on the criteria described by Allen *et al.*,^16^ as follows: (i) peak activation coordinates were in grey matter and had low spatial overlap with known artefacts (vascular, ventricular, motion or susceptibility); (ii) time courses were dominated by low-frequency fluctuations, characterized by a high ratio of power <0.10 Hz to 0.15–0.25 Hz^46^; and (iii) time courses had a high dynamic range (the difference between maximum and minimum power frequencies). Through this process, we identified 31 ICNs which were sorted into 7 functional networks (basal ganglia, sensorimotor, auditory, visual, DMN, attention/cognitive control, cerebellar) based on the spatial maps provided by Shirer *et al.*^47^

For these 31 ICNs, subject-specific time courses (obtained from back-reconstruction, as described above) were detrended (for linear, quadratic and cubic trends), despiked using AFNI’s 3dDespike algorithm (http://afni.nimh.nih.gov/afni) to replace outliers with values calculated from a third-order spline fit to neighbouring clean data points and low-pass filtered using a fifth-order Butterworth filter with a 0.15 Hz cut-off frequency.

### Static functional connectivity

We calculated a 31 × 31 static functional connectivity (sFC) matrix for each subject by measuring the pairwise Pearson correlation coefficient between the subject-specific timeseries of each ICN and applying Fisher’s *z*-transform.

### Dynamic functional connectivity

Recent studies have demonstrated that investigating resting-state connectivity in shorter time windows of tens of seconds can reveal dynamic changes in FC, offering greater insight into functional properties of brain networks.^16,48,49^ We assessed time-varying dFC in this cohort using the methodology described in Spencer and Goodfellow,^50^ which builds on the standard sliding window correlation framework^16^ by including a dimensionality reduction step prior to clustering. We used deep clustering,^51,52^ which consists of autoencoders for dimensionality reduction prior to k-means clustering, as this provides more accurate measurements of state temporal properties in synthetic data than other dimensionality reduction methods or k-means clustering alone.^50^ Autoencoders are a type of artificial neural network which, in dimensionality reduction applications, are trained to copy the input data to the output via a low-dimensional encoding layer.^53,54^ The low-dimensional encoding layer extracts salient features from which the original data can be reproduced via the decoding layers.^52,55^ Sliding window correlations and deep clustering were performed as follows.

**Figure 1:**
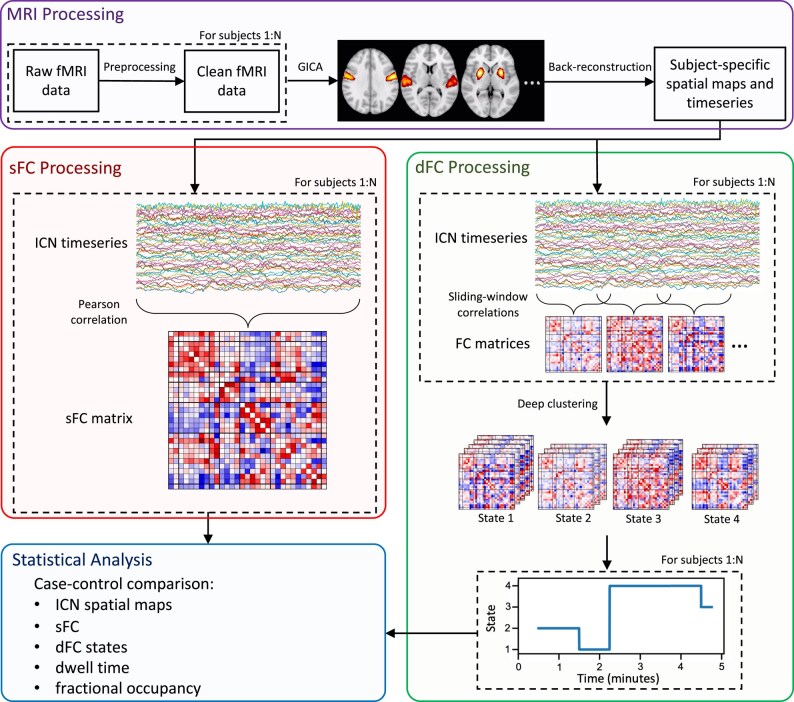
**Pipeline of analysis methods.** Each subject’s resting-state fMRI data was preprocessed, and then, group independent component analysis (GICA) was used to extract intrinsic connectivity networks (ICNs). We identified 31 ICNs and obtained subject-specific spatial maps and time courses using back-reconstruction. Static functional connectivity (sFC) was computed for each subject by measuring pairwise correlation between ICNs. Dynamic functional connectivity (dFC) was computed by sliding window correlations followed by deep clustering^50^ to group FC windows into *k* = 4 states (determined using the elbow criterion of the within-cluster distance to the between-cluster distance). Dwell time and fractional occupancy were measured for each subject. ICN spatial maps and characteristics of sFC and dFC were compared between cases and controls.

First, we used the sliding window correlation approach to convert each ICN time course for each subject to a series of FC matrices, representing time-varying functional connections (Fig. 1). We used a tapered window of length 50 TR (45.3 s), created by convolving a rectangular window with a Gaussian function with a sigma of 6 TR (5.436 s). Sliding the window in steps of 1 TR (0.906 s), we calculated FC within each window by estimating covariance from the precision matrix with L1 regularization,^16,56,57^ where the regularization parameter, λ_L1_, was estimated for each subject using cross-validation, and applied Fisher’s z-transform.

For dimensionality reduction, we used the autoencoder architecture described in Spencer and Goodfellow.^50^ Specifically, this consisted of a fully connected autoencoder with three encoding layers (number of units: 512, 256, 32) and a symmetric decoder. Linear activation functions were used for the low-dimensional layer and output layer, and rectified linear unit (ReLU) activation functions were used for all other layers. We trained the autoencoder for 200 epochs with a batch size of 50, using the Adam optimizer^58^ to minimize the mean squared error (MSE) between the input and output.

We then applied k-means clustering to the low-dimensional representation of the dFC data for all subjects, as follows. First, we selected exemplar FC windows at local maxima in variance and applied 128 repetitions of k-means (max 1000 iterations) to the low-dimensional representation of these windows, each initialized with the k-means++ algorithm.^59^ From these 128 runs, the set of centroids which gave the lowest sum of squared error between each data point and its nearest centroid was used to initialize k-means clustering (max 10 000 iterations) for all windows.

We determined the number of clusters using the elbow criterion of the within-cluster distance to the between-cluster distance, which resulted in *k* = 4. For each subject, we measured the mean dwell time of each cluster (the average time spent in that state) and the fractional occupancy of each cluster (the fraction of the total scan time spent in that state).

### Statistical analysis

After data processing, for each subject we had (i) subject-specific spatial maps for 31 ICNs, (ii) a subject-specific sFC matrix denoting pairwise FC between ICNs and (iii) dFC outputs for each state, consisting of state FC matrices and measurements of dwell time and fractional occupancy.

To investigate differences in ICN spatial maps between cases and controls, we performed case-control comparison of subject-specific spatial maps for each ICN using FSL’s RANDOMISE.^60^ Age and sex were included as covariates in a general linear model, performing two-tailed voxelwise comparison between cases and controls with 10 000 permutations and applying threshold-free cluster enhancement to control the family-wise error rate.

We then investigated group differences in sFC between cases and controls; we regressed age and sex from each pairwise functional connection (pairwise association between ICNs) and performed a two-tailed *t*-test using the residuals. We present uncorrected results, in addition to results after applying false discovery rate (FDR) correction for multiple comparisons.

To compare dFC characteristics, we compared dwell time and fractional occupancy between cases and controls using ANCOVA with age and sex included as covariates. To assess group differences in state FC matrices between cases and controls, we calculated subject-specific state FC matrices as the median of FC windows assigned to each state for a given subject. We performed element-wise comparison between cases and controls by first regressing age and sex from each functional connection and then performing a two-tailed *t*-test using the residuals. We applied FDR multiple comparison correction.

## Results

### Participant demographics

Fifty cases and 43 controls were recruited for the CoolMRI study. Seven cases and four controls did not want to undergo scanning, and seven cases had incomplete data due to movement during the scan. Quality control of the fMRI data resulted in rejection of 13 cases and 19 controls. One additional case was rejected due to poor quality of their T_1_-weighted image, meaning that the data could not be spatially normalized. This left 22 cases and 20 controls with suitable data. Participant demographics are shown in Table 1. There was no significant difference between cases and controls in age, sex, deprivation index or framewise displacement. As previously reported,^29,30^ cases had lower cognitive scores (*P* = 0.0053) measured by the Wechsler Intelligence Scale for Children 4th Edition,^61^ and a larger proportion of the case group were at risk of motor impairment (*P* = 0.0221), defined as a score under the 15th centile on the Movement Assessment Battery for Children 2nd Edition (MABC-2).^62^

**Table 1:** Participant demographics and perinatal clinical information

	Cases (*n* = 22)	Controls (*n* = 20)	*P*
Age, median (IQR)/years	7.08 (6.85–7.52)	6.75 (6.48–7.25)	0.0909
Sex, *n* male (%)	7 (32)	11 (55)	0.2118
Deprivation index, median (IQR)	6 (4–9)	7 (5–8)	0.4099
Framewise displacement, mean ± standard deviation/mm	0.302 ± 0.090	0.274 ± 0.083	0.3077
**Cognitive and motor scores**			
Full-scale IQ, median (IQR)	98 (89–103)	108 (99–116.5)	0.0053
MABC-2 total score, median (IQR)	11 (6–13)	11 (9.5–13)	0.4707
MABC-2 score <15th centile, *n* (%)	8 (36)	1 (5)	0.0221
**Perinatal clinical information**			
Assisted ventilation at 10 min of age, *n* (%)	15 (68)		
Cardiac compressions required, *n* (%)	4 (18)		
Apgar score at 10 min of age, median (IQR)	6 (5–7)		
Worst pH within 1 h of birth, median (IQR)	6.98 (6.90–7.13)		
**Amplitude-integrated EEG abnormalities prior to TH, *n* (%)**			
Moderate	21 (95)		
Severe	1 (5)		

Apgar score is measured on a 1–10 scale where a higher score indicates healthier (7–10 indicates good health). Perinatal asphyxia is characterized by pH < 7.20.

### Intrinsic connectivity networks

**Figure 2:**
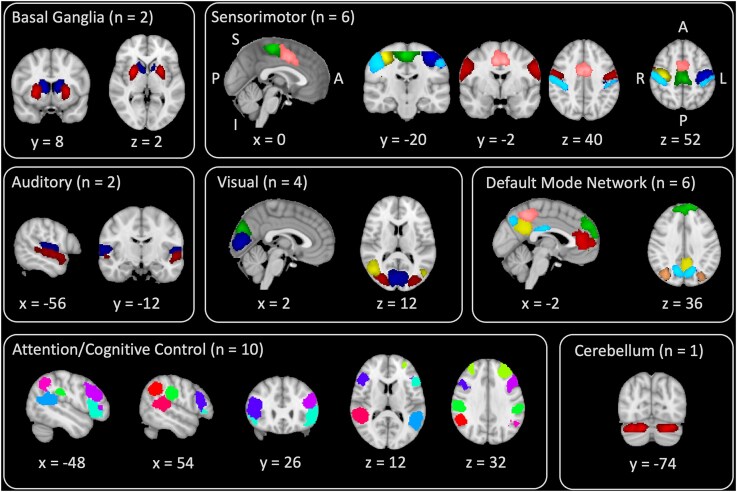
**Spatial maps of intrinsic connectivity networks.** Intrinsic connectivity networks identified by group independent component analysis are grouped into functional networks, with arbitrary colours for visualization. Orientation is indicated by the labels in the sensorimotor panel as follows: S/I, superior/inferior; A/P, anterior/posterior; R/L, right/left.

Figure 2 shows the spatial maps of the 31 ICNs identified from independent component analysis, grouped into 7 functional networks.^47^ These ICNs are consistent with those found in previous studies of children^17,63-65^ and adults.^16,47,66,67^ Details of each independent component are provided in Supplementary Table 1, with spatial maps shown in Supplementary Figs. 1 and 2. There were no case-control differences in ICN spatial maps (*P* > 0.05), indicating the spatial extent of independent components is consistent between groups.

### Static functional connectivity

**Figure 3:**
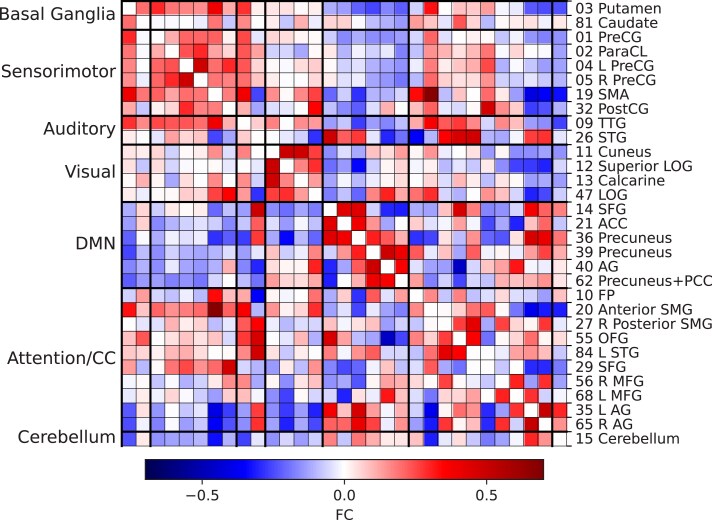
**Average static functional connectivity (sFC) matrix for the whole cohort (*n* = 42).** Independent component number and label is shown in the right, corresponding to the intrinsic connectivity networks (ICNs) shown in Supplementary Table 1. ICNs are arranged into seven functional networks shown on the left. Pairwise functional connectivity (FC) is indicated by the colour bar. The number of each component corresponds to the independent component number in Supplementary Table 1 and Supplementary Figs. 1 and 2. R/L, right/left; PreCG, precentral gyrus; ParaCL, paracentral lobule; SMA, supplementary motor area; PostCG, postcentral gyrus; TTG, transverse temporal gyrus; STG, superior temporal gyrus; LOG, lateral occipital gyrus; SFG, superior frontal gyrus; ACC, anterior cingulate cortex; AG, angular gyrus; PCC, posterior cingulate cortex; FP, frontal pole; SMG, superior marginal gyrus; OFG, orbitofrontal gyrus; MFG, middle frontal gyrus.

The average sFC matrix for the whole cohort is shown in Fig. 3. Similar to previous studies,^16,17^ sFC patterns in this cohort show modular organization, with most functional networks (e.g. sensorimotor, visual, DMN, attention/cognitive control) exhibiting positive connectivity between ICNs within the network. The ICNs which comprise the DMN exhibited negative correlation with most other functional networks.

**Figure 4:**
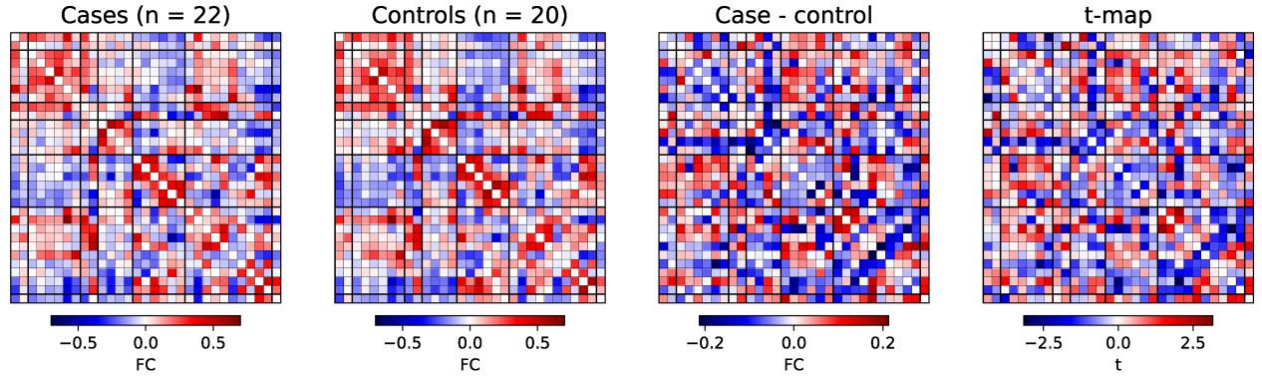
**Case-control differences in static functional connectivity (sFC).** Colour maps from left to right show average sFC in cases (*n* = 22), average sFC in controls (*n* = 20), the difference between these and the *t*-statistic from a two-tailed *t*-test of residual functional connectivity (FC) after regressing age and sex. A *t*-statistic of |*t*| > 2.02 corresponds to uncorrected *P* < 0.05. None of these differences were significant after FDR correction.

We investigated group differences in sFC between ICNs after regressing age and sex. After FDR correction, there were no case-control differences in sFC. The uncorrected *t*-statistic map is presented in Fig. 4. Before multiple comparison correction, there were group differences in FC within the attention/cognitive control network and between this and other functional networks (Fig. 4).

### Dynamic functional connectivity

**Figure 5:**
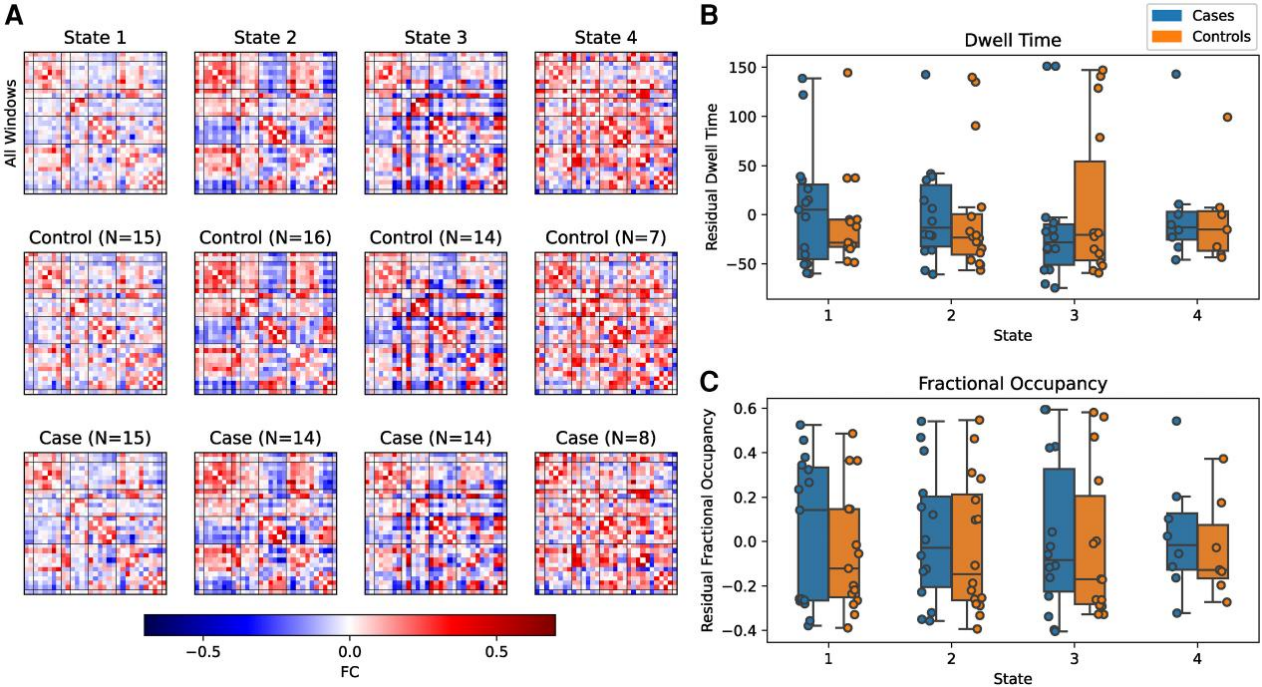
**Dynamic functional connectivity (dFC) state maps and temporal properties.** (**A**) dFC state maps, ordered by prevalence, are shown for the whole cohort (top row) and for controls (middle) and cases (bottom). The distribution of residual dwell time (**B**) and fractional occupancy (**C**), after regressing age and sex, is shown as box plots with boxes indicating the interquartile range with a line for the median and whiskers extending to the range of the data, not including outliers. Individual data points are shown as circles. Dwell time and fractional occupancy were compared between cases and controls for each state using ANCOVA with age and sex included as covariates (*n* shown in panel **A**). There were no significant differences (*P* > 0.05) for dwell time (*t* = 0.633, 0.117, −0.635 and 0.242 for states 1, 2, 3 and 4, respectively) or fractional occupancy (*t* = 0.715, 0.408, 0.243 and 0.447 for states 1, 2, 3 and 4, respectively).

We used sliding window correlations and deep clustering to identify *k* = 4 repetitively occurring FC states, shown in Fig. 5 along with the distribution of residual dwell time and fractional occupancy of each state in cases and controls after regressing age and sex. State 1, which makes up the largest proportion of FC windows, is characterized by very weak connectivity among most ICNs. Previous studies have found similar connectivity patterns in the most frequently observed state and have suggested this may be the average of multiple additional states which are not sufficiently distinct or prevalent to be distinguished.^16,18^ State 2 is characterized by positive connectivity within the sensorimotor network, within the DMN and between the sensorimotor and attention/cognitive control networks but negative connectivity between the DMN and other functional networks. State 3 exhibits strong positive connectivity between ICNs in the visual network, and between ICNs in the DMN, but strong negative connections between many ICNs across all networks. State 4 represents a highly integrated state, characterized by positive connectivity between ICNs across all networks. After FDR correction, there were no differences in the state FC matrices. There were no case-control differences in dwell time or fractional occupancy in any of the states.

## Discussion

In this study, we investigated resting-state networks measured from fMRI in children treated with therapeutic hypothermia for HIE, who did not develop cerebral palsy, and controls matched for age, sex and socioeconomic status. There were no case-control differences in ICN spatial maps, sFC between ICNs and dFC states and temporal characteristics. From 100 independent components derived by spatial group ICA, we identified 31 ICNs based on characteristics of the time courses, spatial maps and power spectra. These ICNs correspond to known resting-state networks previously reported in both children and adults.^16-18,47,63-65^ We found no case-control differences in the spatial maps of these ICNs. We investigated sFC by measuring pairwise correlations between ICN time courses over the duration of the scan for each subject. Before multiple comparison correction, there were case-control differences in attention and cognitive networks; however, these were not significant after FDR correction. Using dFC analysis to investigate dynamic fluctuations in resting-state activity revealed *k* = 4 repetitively occurring brain states. There were no differences between cases and controls in dwell time, fractional occupancy or state FC matrices.

There have been few studies of resting-state FC in children with HIE^68^; resting-state networks in children with HIE have previously only been examined in the neonatal period. Jiang *et al*.^24^ investigated resting-state FC in motor networks in neonates cooled for HIE (five mild, eight moderate and three severe, as determined using Sarnat criteria), in comparison with healthy controls at 1–2 weeks of age. They reported reduced FC between primary motor regions in neonates with HIE and case-control differences in FC spatial maps. Tusor *et al*.^25^ reported reduced FC in auditory, somatomotor, visual and default-mode networks in infants cooled for HIE compared with healthy controls. In a retrospective study of neonates with acute brain injury, 27 of whom were cooled for HIE (14 mild, 7 moderate, 6 severe as determined by Sarnat criteria), more severe outcomes were associated with atypical resting-state activity in the basal ganglia, frontoparietal and default-mode networks.^27^ Two-year follow-up in the same cohort confirmed associations between basal ganglia resting-state activity and motor tone and between the frontoparietal networks and developmental delay, in addition to revealing associations between the default-mode network and both developmental delay and motor tone.^28^ Additionally, Li *et al*.^26^ found that functional brain networks in neonates with severe HIE had lower local efficiency and clustering coefficient compared with those with moderate HIE at around 2 weeks of age, indicating reduced capacity for segregated functional processing. However, the authors did not report whether participants received therapeutic hypothermia and it is not standard care nationwide in China, where the study was carried out.^69^ Our cohort did not include those with cerebral palsy and thus is not directly comparable with the previous studies on infants too young to rule out a diagnosis of cerebral palsy. Our cohort was almost entirely made up of those with moderate HIE (only one case had severe HIE); it is possible that a cohort made up of cases with severe HIE might have more distinguishable differences in FC. However, in the same cohort with a similar proportion of severe versus moderate HIE, we previously reported widespread alterations to structural connectivity and white matter diffusion properties.^29^

The limited case-control differences in ICN spatial maps, sFC and dFC characteristics between the case groups and matched healthy controls are despite previous findings in the same cohort showing widespread alterations to brain structure and white matter connectivity, which are associated with cognitive and motor impairments in cases.^7,29-33^ This may be due to the small sample size; our previous work has identified heterogeneity in the severity of impairments to brain structure and cognition in this cohort^5,29,33^; therefore, any alterations to resting-state brain activity are also likely to be heterogeneous. It may be possible to detect such heterogeneous differences using a deep learning approach for FC ‘fingerprinting’.^70,71^ The subtle differences shared across the cohort would require a large sample size to distinguish from healthy resting-state activity. Before multiple comparison correction, there were group differences in FC between the attention/cognitive control network and the sensorimotor and visual networks (Fig. 4). This may reflect neural correlates of the attention and visuospatial processing difficulties observed in behavioural studies in this cohort^6^ and the altered structural connectivity to regions associated with attention and visuospatial processing previously reported.^29^ However, further study with a larger sample size is required to robustly identify these differences. Differences in brain activity in this cohort may also be detected by a task-based fMRI paradigm which demands the specific aspects of cognition known to differ between cooled children and controls.^72^

It is possible that the minimal group differences reflect a level of recovery of healthy resting-state brain function, despite the structural differences in this cohort. This may suggest that healthy cognitive function could also be recovered in this developmental period. For example, if the appropriate support or intervention was provided in this developmental period between infancy and early school-age, it may be possible to minimize cognitive impairments.^73-76^

### Strengths and limitations

To our knowledge, this is the first study to investigate resting-state FC in children with HIE beyond the neonatal period and the first to investigate dFC in any children with HIE. The main difficulty when scanning children of this age group is movement during the scan, which can affect FC measurements.^77-79^ We took steps to alleviate the effect of movement, using thorough preprocessing and data cleaning procedures to identify and regress noisy signals and motion parameters, in addition to rejecting participants based on quantitative evaluation of movement during the scan. As a result, there was no group difference in framewise displacement. However, rejection of those with excessive movement resulted in a small sample size, which is the main limitation of this study, possibly resulting in type 2 errors. A further limitation is the relatively short duration of the resting-state scan compared with previous resting-state FC studies, at 4 min 32 s (300 volumes). This was done in order to minimize the total scan time and reduce the possibility of movement; however, it may reduce the sensitivity to subtle connectivity differences.

## Supplementary Material

fcae154_Supplementary_Data

## Data Availability

The data that support the findings of this study are available from the corresponding author, upon reasonable request. The code used for dFC analysis (including sliding window correlations and deep clustering) is available at GitHub (https://github.com/apcspencer/dFC_DimReduction).^50^
